# A cluster randomized controlled trial of the be the best you can be intervention: effects on the psychological and physical well-being of school children

**DOI:** 10.1186/1471-2458-13-666

**Published:** 2013-07-17

**Authors:** Martyn Standage, Sean P Cumming, Fiona B Gillison

**Affiliations:** 1 Department for Health, University of Bath, Bath BA2 7AY, UK

**Keywords:** Motivation, Health, Well-being, School, Communication, Self-determination theory, Children, Adolescent, Modifiable risk factors

## Abstract

**Background:**

The ‘Be the Best You Can Be’ (BtBYCB) program is a school-based intervention designed to foster positive physical, psychological, and social development via empowering young people to take ownership over their own personal development. The aim of this work is to determine the effectiveness of the BtBYCB program on (i) pupils’ well-being, self-perceptions, self-esteem, aspirations, and learning strategies; and (ii) changes in modifiable health-risk behaviors (i.e., physical activity, diet, smoking, and alcohol consumption).

**Methods/design:**

A two-arm cluster randomized controlled trial employing a wait-list control plus qualitative and mixed-method evaluations was used. Participants were school pupils from Years 7 and 8 (aged 11–13 years). Ten schools located in southwest England were randomly allocated to receive the BtBYCB intervention (n = 5 schools; 711 pupils) or a control condition (i.e., usual Personal, Social, and Health Education classes) (n = 5 schools; 622 pupils). Participants in the intervention condition received a program consisting of (i) a talk from an Olympian/Paralympian; (ii) 11 one-hour teacher-led PSHE classroom sessions in which pupils identified their aspirations, values, and interests and explored and acted on these via activities such as personal development planning, goal-setting, and peer-mentoring; and (iii) participated in a celebration event (e.g., second visit from Olympian/Paralympian and short individual presentations). Data were collected at baseline, post-intervention, and at 3-month follow-up.

Focus groups (pupils and teachers) and individual interviews (headteachers) were conducted in the 5 intervention schools to (i) gain an in-depth understanding of mechanisms of change; (ii) explore ways in which the participants’ motivation and engagement could be enhanced, and (iii) elicit user-feedback pertaining to how the program, content, and appeal could be improved.

A mixed-method approach was used to describe and explain the differing experiences of particular groupings within and across the intervention schools; i.e., those for whom the program was effective, those that experienced little, if any change, and those for whom the program led to an inverse effect.

**Discussion:**

The findings of this work will provide insight into the effectiveness of an innovative and child-centered program. The research will inform improvements to the BtBYCB program as well as other interventions targeting child/youth health and wellness.

**Trial registration:**

The trial is registered as Current Controlled Trials ISRCTN99443695.

## Background

The Be the Best You Can Be (BtBYCB) program was launched on the back of the awarding of the *Games of the Thirtieth Olympiad* to the city of London as part of the legacy aimed at inspiring the nation’s generation of children (i.e., going beyond the buildings and infrastructure provided by the games themselves). Developed and refined by a team of educational experts, education specialists/policy makers, and university scholars, the BtBYCB program was designed to foster positive physical, psychological, and social development by encouraging pupils to take ownership over their own personal development (i.e., becoming successful, creative and resourceful learners who are capable of living safe and healthy lives, and contribute positively to society). Such overarching aims and objectives directly align with key UK education, well-being and health policies and directives e.g., [1-4].

Following a staff-development day designed to enhance the facilitation skills of those who will be leading the program, the intervention commences with an inspirational ‘launch’ , facilitated by a talk from an Olympian, Paralympian, or other exceptional achiever. The launch session is intended to provide initial motivation to the pupils by helping them to understand what it feels like to be successful, the journey of personal growth involved, and the skills and dedication needed to achieve one’s dreams. The program continues with 11 one-hour teacher-led classroom sessions which are scheduled to occur within Personal, Social, and Health Education (PSHE) (although the topics can be linked across the curriculum in classes such as Physical Education). In these sessions, pupils are challenged to identify their aspirations, values, and interests and through activities such as *personal development planning*, *peer*-*mentoring*, and *self*-*coaching*, raise their awareness and develop the learning, self-management, self-reflection, and interpersonal skills necessary to support their identified ambitions, goals, and objectives. The program culminates with a final session (i.e., the ‘celebration’) in which the pupils are invited to deliver short group presentations to an audience consisting of fellow pupils, school staff, and invited guests (e.g., the guest speaker, parents, and members of the community). This presentation provides the pupil with an opportunity to reflect upon their personal achievements, share with others what the program meant to them, and reflect on the life-skills developed through the intervention. To facilitate the delivery of the program, each school has a BtBYCB team consisting of year tutors, teachers, PSHE staff, and a senior staff member.

With the intention of changing how pupils behave, view success, function, and develop skills for lifelong learning and health it is clear that an understanding of the motivation of pupils to engage and persist in the intervention is fundamental to the success of BtBYCB program. A general theory of human motivation that addresses the quality of motivation as well as the explicit conditions that promote optimal engagement, growth, health and development is *self*-*determination theory* (SDT; [5,6]). SDT is the product of a systematic and comprehensive program of inductive research spanning the past five decades, and proposes that the nature and focus of motivation that gives rise to action can vary greatly, and it is this variation in motivation quality that serves to predict the degree to which behavior is sustained, active, and effortful. SDT also provides explicit insight into how to foster improvements in (i) autonomous motivation and (ii) physical and psychological health. Broadly speaking, within SDT social conditions supportive of a person’s experience of three basic psychological needs for *autonomy*, *competence*, and *relatedness* are argued to foster the most volitional and high quality forms of motivation. This in turn promotes engagement with activities, including enhanced performance, persistence, and creativity [5,6]. In contrast, SDT proposes that the degree to which any of these three basic psychological needs are unsupported or thwarted within a social or cultural context, will relate to a measurable negative impact on an individuals’ quality of motivation and wellness (e.g., ill-being, passive engagement, and restricted development) [6].

The BtBYCB program is aligned with tenets embraced within SDT. To this end, SDT postulates that autonomous types of motivation result in positive consequences (e.g., optimal functioning, behavioral persistence, effortful engagement, eudaimonic and hedonic well-being), whereas controlling types of motivation lead to negative outcomes (e.g., school drop-out, elevated anxiety, and greater experiencing of ill-being). Considerable empirical work in education settings has shown autonomous forms of motivation to lead to a number of desirable outcomes such as academic persistence [7], higher quality learning [8], improved achievement [9], enhanced well-being [10], and greater levels of school engagement [11]. Parallel findings have been reported in a wide array of contexts including sport, business, health-care, and exercise settings [cf. 12,13].

An appealing feature of the SDT framework is that it provides explicit insight into how to foster increments in (i) autonomous motivation and (ii) physical and psychological well-being. Within SDT a number of malleable antecedents to the satisfaction of the psychological needs that can be readily incorporated into the proposed intervention are specified. For example, prior research demonstrates that to support the need for autonomy, social contexts (e.g., lessons) need to provide choice, promote initiation, and understanding, while minimizing the need to perform and act in a prescribed manner. When the need for autonomy is supported in this manner, autonomous enactment in activities, well-being, learning, behavioral persistence, and adaptive health-related consequences are all enhanced [12]. Such interpersonal social contexts are termed autonomy-supportive environments.

In addition to promoting autonomy-support, in this work we will also target the social conditions necessary to facilitate competence and relatedness. For example, to promote pupils’ perceptions of competence, many activities/tasks will have self-referenced (or task-involving) standards and indicators of improvement associated with them, and feedback will be provided in a similar self-referenced manner [13]. This will provide informational feedback to pupils helping them to learn what is involved in making further improvements and how to notice and acknowledge their progress rather than focusing on their performance relative to other pupils. To facilitate relatedness, certain tasks/activities (e.g., peer mentoring) will encourage small group activities to promote cooperation and reciprocal relationships [13,14].

A second line of research has shown goal content manipulations (i.e., framing “what” pupils may hope to obtain from taking part) presented in the form of text scripts to differentially predict pupils’ responses to desirable education outcomes such as persistence and performance [15,16]. Specifically, previous research has shown adaptive outcomes to be a function of promoting goals that are intrinsic in nature as opposed to those that are directed towards external indicators of worth. As such, in this work the foci will be towards “inwardly-focused” goals such as personal growth, affiliation, community contribution, and maintenance of physical health.

Finally, because certain activities underpinning personal growth, achieving personal excellence, and accruing health benefits are not always appealing (i.e., albeit desirable they can be mundane, repetitive, etc.), an understanding of how to support an individual’s motivation to partake in such activities represents an important aspect of this research application. To this end, a process proposed by SDT (i.e., internalization) provides valuable information as to how to promote adaptive engagement in less appealing activities. Specifically, to facilitate the internalization process, Deci and colleagues e.g., [17,18] have identified the following social conditions; (i) a meaningful rationale expressing why it is important to partake in the activity, (ii) the interpersonal context in which the behavior is performed to be supportive of the basic needs for autonomy, competence, and relatedness so as to facilitate autonomous regulation and integration, (iii) an acknowledgement of the pupils’ feelings and perspective about the activity, and (iv) the use of language that conveys choice rather than control. Again, these principles have been incorporated into the BtBYCB program.

### The present work: Research aims and hypotheses

#### Primary aim

The primary aim of this study is to provide an empirical assessment of the BtBYCB program via the use of a cluster randomized controlled trial (CRCT). Specifically, the following research aims and hypotheses will be addressed in this trial:

### Aims

• To statistically examine whether the BtBYCB intervention leads to increased levels of eudaimonic well-being, hedonic well-being, self-esteem, self-perceptions, and adaptive learning strategies.

• In view of the healthy lifestyle messages embedded in the program, statistically examine whether the BtBYCB intervention leads to desirable changes in reported modifiable health-risk behaviors (i.e., physical activity level, dietary intake, tobacco use, and alcohol consumption). Further, in a subsample we will test whether changes in objectively assessed physical activity levels occur as a function of participating in the program.

• Using (i) qualitative methods; and (ii) a mixed-methods approach, describe, analyze and explain the differing experiences of participants engaged in the BtBYCB program.

### Hypotheses

• Engagement in the BtBYCB intervention will lead to increases in markers of eudaimonic well-being, hedonic well-being, and self-esteem.

• Engagement in the BtBYCB intervention will lead to desirable improvements in reported modifiable health-risk behaviors (i.e., physical activity level, dietary intake, smoking, and alcohol consumption). Further, and in a subsample, we hypothesize that objectively assessed physical activity levels will increase as a function of participating in the program.

• It is hypothesized that pupil improvements on markers of health and well-being will be mediated by increases in their perceptions of an autonomy supportive teaching context, satisfaction of autonomy, competence, relatedness, and also by improved autonomous motivation scores.

#### Secondary aim/objective

In addition to the main CRCT, we will conduct two supplementary elements of work. Combined, these phases of work will provide rich information of the *lived experiences* of participants and provide greater insight and understanding of the program experiences and user needs.

The first segment of work involves focus groups (or individual interviews in the case of headteachers) with participants to explore how the underpinning principles within SDT can be/have been integrated into the BtBYCB program. This aspect of work will also build an in-depth understanding of the “active ingredients” of the program, will explore ways in which the participants’ need-satisfaction and motivation could be further enhanced, and look into ways in which the user-group (i.e., pupils, teachers and headteachers) think that the program and its content and appeal can be improved.

The second component of work pertains to a simultaneous mixed-method approach encompassing qualitative and quantitative methodologies. Specifically, this approach will go beyond the ‘elaboration’ stage used in the focus group work to a ‘complementary’ perspective cf. [19]. Indeed, the purpose of the mixed-methods element of this study is to gain an in-depth account of the differing experiences of the program for particular groupings. The quantitative data will be used to identify pupils in each of the five intervention schools with different experiences of the intervention (i.e., those for whom the program was most effective, those who gained little, if any, change from the program, and those for whom the program had inverse effects). Informed by the quantitative data (thus, a QUAN + qual notation), this approach will seek to gain an in-depth account of the program experiences of participants with contrasting profiles. This approach will provide an enriched account of the data in a synergetic fashion as quantitative methods are suited to specifying relationships and qualitative for explaining and understanding relationships [20].

## Methods/design

### Ethics

This research was approved by the University of Bath School for Health Research Ethics Approval Panel (ref. EP 08/09 45) and is registered with Current Controlled Trials (registration number: ISRCTN99443695).

### Design

A single center CRCT employing a wait-list control group, supplemented by focus groups and a mixed methods process evaluation. The unit of randomization was school. Individual pupils will be the unit of analyses, yet we will take into account the nesting effects of schools and classes.

### Participants and setting

Secondary school children aged between 11 and 13 years attending 10 schools in the southwest of England served as participants in this project. Following ethical approval, parental consent, and participant assent, 1333 pupils were recruited for the CRCT using a cluster sampling approach. Pupils were randomly assigned by their school to either (i) BtBYCB intervention (n = 711 pupils from 28 classes) or (ii) a control group receiving standard PSHE lessons (i.e., standard care; n = 622 pupils from 30 classes). For the secondary analyses, a randomly selected sample of pupils and teachers were selected from each of the five intervention schools (n = 5). For the mixed-methods element, participants in each of the five schools were stratified by outcomes using a tertile split so as to acquire a purposeful sample encompassing different pupil experiences of the BtBYCB intervention (8 participants per grouping in each of the 5 intervention schools).

### Inclusion/exclusion criteria

All Year 7 and 8 school pupils in classes at the participating schools were eligible to participate in the program and the associated research. An inability to be involved in the intervention sessions was the only exclusion criteria (i.e., based on the opinion of the participating school).

### Sampling and randomization

To reduce contamination effects and bias, school was used as the randomization unit in this work. Allocation to either the intervention or the control arms of the trial occurred after schools had been recruited. A block randomization (1:1 ratio) was performed by an independent agency (i.e., in this case a trained individual within the SouthWest Regional Development Agency) and the balance between the trial arms was based on the following strata: socioeconomic status, urban/rural, and small/large. In agreeing to partake in this work, the school’s senior management had to be willing to accept the randomization status of the school. Potential disappointment with being allocated to the control condition was offset by the use of a wait-list control.

### Sample size calculation

Using a sample size calculator software package [21], the 58 classes used in this work based on the average size of class (i.e., 25 pupils; [22]) permitted the detection, at the 5% significance and 90% power level, of a standard difference of 0.25 (i.e., .25 of a standard deviation can be detected with an intra-cluster correlation (ICC) of .05). In recognizing that some classes were smaller than the average 25 pupils, a more conservative estimate using the same criteria (5% significance with 90% power assuming an ICC of .05) showed that .27 of a standard deviation could be detected with 50 classes with 25 pupils in each cluster. The current sample size also accommodates analyses at school and classroom levels (i.e., pupils are nested within classes which are, in turn, nested within schools).

### Intervention and implementation

The BTBYCB program consists of a launch event (i.e., inspirational talk), 11 teacher led classroom sessions that are embodied and aligned with the aims and intent of the new Secondary curriculum, and a concluding Olympic celebration event. Adopting a cross curricular approach (i.e., sessions can be delivered across PE, PHSE, and other curriculum areas), the program’s aims are to (i) inspire pupils to engage with the Olympic ethos and values; (ii) help pupils gain greater self-awareness and self-responsibility; (iii) empower pupils through provision of self-regulatory skills; (iv) raise pupils aspirations and expectations for success, and (v) provide pupils with the skills, resources, and insights to face the challenges of lifelong learning and fulfillment.

As part of the BtBYCB program, the school teaching staff responsible for delivery attend a day long teacher training workshop prior to implementation of the program. These sessions are delivered by the developers of the program and held separately for each school. The training sessions include explanations of the program aims, structure and content, an introduction to the program curriculum and accompanying workbook, and lessons and advice on how to best deliver each classroom session. Teaching staff attending the teacher training session all receive a BTBYCB training manual that includes learning materials, guidelines, and instructions on how to deliver each session effectively.

In total, the BTBYCB program consists of 13 sessions. The program commences with an introductory talk and question and answer session delivered by an Olympian, Paralympian, or other high achiever. This session is designed to help pupils experience life through the eyes of a high achiever (i.e., appreciate their inner perspective), and discover the personal journey and skills required to succeed in life. The concept of Olympic and/or Paralympic achievement is then extended to the pupils, encouraging them to establish personal goals that represent success in life, and not just sport-related activities.

The eleven BTBYCB classroom sessions consist of Olympic and Paralympic themed lessons, exercises, and activities that provide pupils with the knowledge and skills necessary to realize and raise their aspirations, and ‘ask the right questions’, raising personal and social awareness. As each pupil progresses through the program they create a personal success map that records how they have realized and taken ownership of their aspirations, and created opportunities and strategies to help in achieving these aims (i.e., the aim is to enhance awareness and responsibility of self-endorsed goals and motives). The program culminates with an Olympic/Paralympic celebration, in which pupils perform a short presentation to an audience of their peers, teachers, and invited guests (e.g., the guest speaker, parents, and members of the community) on what the program has meant to them. Table 1 provides an overview of the sessions within the BtBYCB program.

**Table 1:** Overview of the BtBYCB program session structure

**Session**	**Content and purpose**
1	Olympian Launch: An Olympian/Paralympian is used to inspire and motivate pupils through the story of his or her personal journey, making pupils aware of the skills, values, and work required to succeed.
2.	Visioning: Pupils reflect upon what inspires and interests them, and are encouraged to autonomously develop a vision of their future, visualizing the possibilities available to them and how they might achieve them (i.e., increasing their self-awareness).
3.	Coaching & Mentoring Skills (1). Through discussing what it means to be an effective coach or mentor and the difference between open and closed questions, pupils begin to understand the principles of self-awareness, self-regulatory processes, and responsibility and the power of effective questioning.
4.	Coaching & Mentoring Skills (2). Pupils develop and apply their questioning skills using the GROW (Goals, Reality, Options, & Will) model as a means for constructing powerful conversations regarding their future aspirations. The development of peer mentoring skills is also a focus of this session.
5.	Coaching & Mentoring Skills (3). Through group conversations pupils explore the skill and value of empathic and attentive listening, generating valuable feedback insight and support in relation to their aspirations. This session helps the pupils to take on board, and respect, the views and values of others.
6.	The Power of Alignment: Pupils develop the concept of leading a balanced lifestyle (i.e., mind, body & spirit) and are encouraged to develop a positive self-image, identifying personal strengths and planning future steps.
7.	Body - Health and Fitness: Pupils identify the factors that affect human performance, personal health, and well-being, and discuss their current reality against these factors, prioritizing personal improvements (i.e., actions, measures, and outcome).
8.	Using the Mind Effectively: Pupils explore learning strategies that use the mind for maximum effect (e.g., multiple intelligences, mind mapping, brain gym), with the purpose of developing new skills and way of thinking. This session also covers autonomous decision making.
9.	Emotions – Enhancing Relationships: Pupils identify the values and emotions that underpin their behavior and how these factors impact their relationships with others.
10.	Understanding and Harnessing the Olympian Spirit: Pupils explore the value of prosocial behaviors and how each individual can make a positive contribution to society. They identify with the value and enjoyment of everyday life and recognize the role of significant others in making positive contributions to their own lives and the lives of others.
11.	Olympian Responsibility Through Teamwork: Through group activities pupils explore the qualities that characterize high performing teams and recognize the benefits of working as an effective team.
12.	Olympian Reflection: Pupils review their work through group presentations, sharing success stories, personal aspirations, and future success maps.
13.	Olympic Celebration: Through group presentations to teachers, peers, and invited guests (e.g., the guest speaker, parents, and members of the community), pupils have the opportunity to share and demonstrate their personal progress, successes to date, and future aspirations.

### Control group

Participants in the control group attended their usual PSHE classes (i.e., they received standard care). In the main phase of the trial, the participants provided data pertaining to the study variables of interest. The measurements in the control schools were scheduled for the same time points as for the intervention group.

### Data collection

Because the participants in the proposed work were under the age of 16 years, written approval for the program evaluation was sought from headteachers who were asked to act in loco parentis. Letters were posted to all parents providing information about the study and seeking consent. To this end, only those who did not wish their child to take part were required to respond (i.e., passive content was sought). Participants also provided written consent and were informed that they could withdraw from the study at any time, without incurring any negative repercussions; the program would be delivered to all pupils in the class but participation in the evaluation research was optional.

Accelerometers (Actigraph GT1M) were issued to a subsample of pupils from the intervention and control schools (n = 103). The accelerometers were secured to the participants’ waists via an elastic belt and positioned on the midaxillary line on the right hip. Participants were provided with verbal and written instructions regarding accelerometer use and asked to wear the device each day (from waking) before removing it at night for sleep. Participants were also asked to remove the device when bathing, showering, or engaging in water based or contact sports. Participants were asked to wear the accelerometer for a period of 8 days and the devices were set to record data in 60 second epochs.

Self-report questionnaires were completed during school hours as part of a series of supervised classroom exercises. Sessions were supervised by trained research assistants and members of the schools teaching staff (e.g., headteachers, teachers, and learning support staff). Instructions for completing the questionnaires were read out to the pupils in advance of the session. Pupils were asked to raise their hand if they had any questions regarding the nature or content of the questionnaires. Members of the research team were present during the sessions to assist the participants.

During the questionnaire sessions, small groups of participants were taken to a separate room for measurements of height and weight (i.e., to avoid potential embarrassment). Height and weight were measured using standardized procedures [23]. Participants were required to remove their shoes for assessments of height and weight. Assessments were performed on an individual basis and pupils were not told their weight. Chronological age in decimals was calculated as the difference between each participant’s date of birth and date of measurement. Biological parents of the participants also provided their heights via self-report. As adults generally overestimate height, the self-reported height of each parent was adjusted for over estimation using an equation constructed from over 1000 measured and estimated heights of adults [24].

### Primary and secondary outcomes

#### Primary outcomes

*Eudaimonic well*-*being* refers to *living well* (e.g., aspiring to personal excellence, pursuing a meaningful life, contributing to the lives of others, holding intrinsic values, etc.). Changes in eudaimonic well-being were assessed both at the contextual and life-domain levels of generality cf. [25]. Because SDT provides explicit information regarding the conditions required to promote *eudaimonic well*-*being*, we also examined whether the intervention achieves its aim of promoting significant changes in a lifestyle supportive of personal growth, through facilitating need-satisfaction and autonomous enactment. While eudaimonia refers to a lifestyle, the content of the BtBYCB program is also likely to directly (and indirectly through eudaimonic well-being) foster changes in the more temporal experiences of positive well-being. These are reflective of *hedonic well*-*being* or subjective well-being, indexed by life satisfaction, the presence of positive affect, and absence of negative affect cf. [26], and were also recorded as primary outcomes. Finally, we also assessed changes in the participants’ reported level of self-esteem, self-perceptions, and patterns of adaptive learning strategies.

#### Secondary outcomes (health-related)

The content of the BtBYCB program embraces messages pertinent to pursuing a healthy lifestyle and encompasses activities that require pupils to evaluate, reflect, and take ownership of improving their health status. As such, in the present work we also tested whether the intervention promoted positive changes in reported levels of a number of modifiable health-risks; namely physical activity level, dietary intake, tobacco use, and alcohol consumption. In addition to direct changes, it is likely that changes will be mediated by increases in eudaimonic well-being as this index of well-being has been positively linked with physical health and mortality [27]. Finally, pupils within a randomly-selected subsample of school classes balanced across the conditions wore an accelerometer to objectively assess changes in weekly physical activity level at one week before and after the intervention, and at 3-month follow-up (n = 103 pupils). Body-mass index (BMI) was also obtained from most participating pupils and will be expressed as age-adjusted.

### Measures

All primary and secondary outcome measures were assessed at baseline (Time 0), at the end of the intervention (Time 2), and at 3-month follow-up (Time 3). Data pertaining to theoretically-relevant variables [cf. 5,6] that may mediate changes were assessed at a mid-point data collection (week 6 of the program). The measures selected in the trial were:

#### Primary

*Eudaimonic well*-*being* was assessed at the contextual level by the Personally Expressive Activities Questionnaire [28] and at the life-domain level by items from previously validated questionnaires (i.e., the Personal Growth subscale [29], Prosocial subscale of the Strengths and Difficulties Questionnaire [30], Subjective Vitality Scale [31], and the Proactive Attitude Scale [32]. *Hedonic well*-*being* was assessed by the Positive and Negative Affect Scale for Children [33] and Life-Satisfaction in Adolescents Scale [34]. *Self*-*esteem* was assessed by Rosenberg’s scale [35], and *adaptive learning strategies* by the Patterns of Adaptive Learning Scale [36].

#### Secondary

Self-reported *physical activity levels* were assessed by the Physical Activity for Older Children and Adolescents Questionnaire (PAQ-A; [37]). *Smoking* and *Alcohol Intake* were assessed by subscales from the CDC’s Youth Risk Behavior Survey [38], while *dietary intake* was measured via the Food Frequency Questionnaire [39]. Additionally, *weight* and *height* (to provide Body Mass Index scores), and *objectively assessed physical activity level* data were obtained from a subsample of 103 participants. With physical activity in mind, data were collected using the ActiGraph GT1M unit. This unit has been validated in child and adolescent populations (e.g., [40]). The ActiGraph has no external controls or display, thus preventing user-feedback from influencing behavior.

#### Mediating variables

To explore the psychological processes underpinning the study hypotheses, the stems of the following inventories that have been successfully used in school-settings were slightly amended to target the BtBYCB program (intervention group) and PSHE (control condition): Perceptions of *Autonomy*-*Support*[41], *Basic Need Satisfaction* (viz., for autonomy, competence, and relatedness) [42], *Self*-*Regulation Towards Learning*[43], and the activity *Value*/*Usefulness*, *Choice*, *and Interest*/*Enjoyment* subscales from Intrinsic Motivation Inventory [44].

#### Moderating variables

Gender, age, biological maturation, SES, ethnicity, and access to facilities affect adolescent health behaviors and well-being. Thus, these variables were recorded and will be controlled for in the analyses.

### Supplementary research and methodologies

In addition to the primary research methodology (viz., the CRCT), two additional phases of work were conducted. These additional elements of research were:

#### Qualitative component

Pupils were randomly recruited from the intervention schools of the CRCT to partake in focus groups (i.e., 5 focus groups with 5 pupils in each). The main aims of this aspect of the work were to: (i) build an in-depth understanding of the “active ingredients” of the BtBYCB program that facilitate autonomy, competence, relatedness, and autonomous engagement with the intervention, (ii) investigate ways in which the participants’ need-satisfaction and motivation could be further enhanced, and (iii) explore ways in which the pupils think the program and its appeal could be improved. Across the intervention schools, five focus groups (involving 4 teachers in each school) and individual interviews (i.e., 5 × 1 headmaster per school) were also conducted so as to solicit views on the existing teacher education module, including (i) any difficulties experienced, (ii) whether any elements were not convincing, and (iii) ways in which the program could be better translated to practice (e.g., gaps in provision, content, etc.).

All participants were encouraged to talk openly about their experiences and the focus group content was transcribed verbatim and coded. As the focus group format can facilitate the recollection of experiences and thoughts (i.e., the input of one participant can stimulate the input of another) and for some the presence of others may inhibit a contribution to the process, a sample of the participants were randomly selected to respond to a brief summary of the emerging themes to result from the focus groups and asked to comment on, expand, and clarify the findings in *one*-*to*-*one* interviews cf. [45]. New data emerging from the individual interviews will also be subjected to further content analysis, and thematic extraction conducted cf. [46].

#### Mixed-methods component

The sample-frame for the mixed-methods component of the work consisted of participants that participated in the CRCT. Via a tertile split, participants were separated into three groups based on whether the program (i) was effective – i.e., a marked increase in reported scores were listed pre- to post; (ii) had no change – i.e., no noticeable change from pre- to post; and (iii) was counterproductive – i.e., a group for whom the program had an inverse effect pre- to post-. Participants meeting one of these criteria were short-listed, and participants selected from the list at random until the quota of interviewees was met. A total of 8 pupils per group were interviewed in each of the schools that received the BtBYCB intervention. The interviewer was blinded to the groupings to reduce bias. Semi-structured interviews were conducted to explore the process of change behind the responses of participants, and specifically, to extract potential triggers to change.

### Data Analyses

Initially, analysis of variance (ANOVA) will be conducted to establish significant differences for the primary and secondary outcome variables between the two trial arms (allowing for the effects of nesting). Effect sizes, confidence intervals and confidence levels will be calculated. Changes from baseline in the primary and secondary end-points will be analyzed using an analysis of covariance (ANCOVA) model with the intervention as the main factor and baseline as a covariate. Further, multilevel modeling (using HLM 7 software; [47]) will be used to assess the strength of the effect of the intervention on the outcome variables (both primary and secondary) when controlling for nesting effects. Class and school will represent higher order units of analysis, and individual responses the lower order unit of analysis. In such analyses, it is possible to enter class and/or school as a random effect and all other variables as fixed effects.

For the focus groups and individual interviews, the recordings will be transcribed verbatim and analyzed using interpretive phenomenological analysis [48]. Statements will be coded by the investigators and a research assistant with themes within and across participants being extracted. Dependability and trustworthiness of the analysis will be demonstrated through; (i) researcher triangulation (i.e., discussion between researchers to establish all inferences are supported by the data), (ii) the triangulation of qualitative with quantitative data [49], and (iii) participant debriefing to check the researcher’s interpretations, and allow interviewees to make further comments.

## Discussion

Within this paper, a study protocol outlining a robust and systematic empirical assessment of the effectiveness of the BtBYCB intervention on indices of pupil well-being, learning, and health has been described. The protocol described also permits the testing of theoretically-informed mediators designed to form “active ingredients” of the BtBYCB intervention. A strength of framing the work within an empirically supported framework of motivation is that a detailed understanding of how and why the BtBYCB impacts on the primary and secondary outcome variables can be gleaned. That is, the effects of different intervention components can be mapped onto key determinants shown to be robust predictors of high quality forms of motivation as well as indices of adaptive health and well-being [cf. 5,6]. The work is also novel by adding a qualitative and mixed-methods approach to gaining a better understanding of the intervention. That is, the use of the simultaneous mixed-methods approach builds on recent calls for such methods [e.g., 13] and to our knowledge represents the first application of such an approach in the extant SDT-related literature. Such an approach can be readily extended and modified to other areas of study that focus on the mechanisms underpinning adaptive and sustained behavior-change.

There are a number of limitations worthy of note. First, the follow-up period of 3 months is short in duration. Second, many of the measures used were self-report in nature. Thus, issues surrounding common method variance and reporting biases cannot be negated. Future work assessing the BtBYCB program would benefit from using more objective measures across all participants (i.e., we assessed physical activity via accelerometry, but only within a subsample of participants). Future evaluation should also include cost-effectiveness analyses to ascertain whether the intervention leads to meaningful changes within a financially sustainable model.

Findings from this work will be disseminated to practice based audiences (e.g., workshops, practitioner/professional journals, presentations to school-bodies and/or public health departments), lay summaries of findings to the general media (e.g., via newspapers, radio, the internet, and television), and within peer-review articles and conference presentations. The results from this research will be reported akin with the Consolidated Standards of Reporting Trials (CONSORT) recommendations [50].

## Abbreviations

BMI: Body Mass Index; BtBYCB: Be the best you can be program; CRCT: Cluster randomized controlled trial; PSHE: Personal social and health education classes; SDT: Self-determination theory.

## Competing interests

The authors declare they have no competing interests.

## Authors’ contributions

MS and SPC designed the study and obtained the research funding. FBG contributed qualitative expertise in the design. MS drafted the first draft of the manuscript. SPC and FBG provided revisions to the paper. All authors read and approved the final manuscript.

## Funding

This study was funded by the Economic & Social Research Council (ESRC) (UK) ref: RES-177-25-001. The views expressed in this publication are those of the authors and not necessarily those of the ESRC.

## Pre-publication history

The pre-publication history for this paper can be accessed here:

http://www.biomedcentral.com/1471-2458/13/666/prepub
